# Value of the combination of a smartphone-compatible infrared camera and a hand-held doppler ultrasound in preoperative localization of perforators in flaps

**DOI:** 10.1016/j.heliyon.2023.e17372

**Published:** 2023-06-16

**Authors:** Weiwen Zhu, Yi Yang, Jiyong Jiang, Qingtang Zhu, Jian Qi, Bengang Qin, Jingyuan Fan, Ming Fu, Ping Li

**Affiliations:** a Department of Orthopedics, Trauma & Microsurgery, Joint Surgery, First Affiliated Hospital of Sun Yat-sen University, Zhongshan No. 2 Road, Guangzhou, 510080, China; b Fourth District of Microsurgery and Hand Department, Heping Orthopedics Hospital, Jude Nan Road 112-120, Guangzhou, 510305, China

**Keywords:** dynamic infrared thermography, Handheld Doppler ultrasound, Artery perforators, Lateral circumflex femoral artery

## Abstract

This study was conducted to evaluate the effectiveness of the FLIR ONE PRO, a thermal imaging camera for smartphones, combined with handheld Doppler (HHD) in the localization of perforator arteries and to assess the efficacy of the FLIR ONE PRO in distinguishing perforators of the descending branch of the lateral circumflex femoral artery (LCFA) from other perforators of the anterolateral thigh perforator (ALTP) flap. We enrolled 29 free perforator flaps from 22 patients in our study. Before surgery, dynamic infrared thermography was performed using a FLIR ONE PRO to visualize hotspots on the flaps. Subsequently, HHD was used to further determine the perforators under the hotspots, which were ultimately identified and confirmed through intraoperative findings. Additionally, infrared images of the ALTP flap were analyzed using FLIR Tools. The performances of the FLIR ONE PRO and FLIR ONE PRO + HHD groups were evaluated by comparing the intraoperative findings. Using FLIR ONE PRO + HHD, 119 hotspots and 106 perforators were identified during surgery. Using FLIR ONE PRO + HHD, sensitivity and positive predictive value were 97.87% and 88.46%, respectively, in the young (age≤45 years). In the elderly group (age>45 years), these percentages were 93.22% and 82.09%, respectively. In addition, we found that the FLIR ONE PRO could be useful for differentiating perforators in the descending branch of the LCFA from other perforators within 5 min. The results showed a sensitivity of 96.15%, a specificity of 98.9%, a positive predictive value of 96.15%, and a negative predictive value of 98.9%. Compared to using FLIR ONE PRO alone, the combined application of HHD and FLIR ONE PRO had a higher value in perforator localization by increasing the positive predictive value. The FLIR ONE PRO may have significance in the rapid prediction of perforators deriving from the descending branch of the LCFA.

## 1 Introduction

In traumatology and microsurgery departments, perforator flaps are often used to reconstruct extremity defects [1], as this approach has multiple advantages such as the sparing of underlying muscles, reduced donor-site morbidity, decreased operative time, and improved aesthetic outcomes [2]. Six of the most commonly used flaps are the deep inferior epigastric artery flap, superior gluteal medial artery flap, thoracodorsal artery flap, descending branch of the lateral circumflex femoral artery (LCFA) flap, transverse branch of the LCFA flap, and medial sural artery flap [3]. The LCFA flap, also known as the anterolateral thigh perforator (ALTP) flap, is the most commonly used in body defects. The advantages of the ALTP flap are the long vascular pedicle, good vascular diameter, and minimal donor-site morbidity [4]. This flap can be used as a pedicled, transposition, or free flap. Successful design and transplantation of a free flap mostly rely on a robust blood supply from the perforators [5]. Thus, the preoperative mapping of perforators remains a challenge for flap surgery.

Currently, various approaches are applied in the preoperative localization and selection of perforator arteries [6], such as digital subtraction angiography [7], computed tomography angiography, and magnetic resonance angiography [8]. However, because of their disadvantages, such as their high cost, invasiveness, and requirement of large devices, these methods might not be the first choice when performing regular examinations for perforator detection. Color Doppler ultrasonography (CDU) becomes a widely used method for preoperative perforator prediction that provides highly sensitive and predictive information about the emerging points, courses, and blood-flow characteristics of cutaneous vessels [9,10]. However, the requirements of a skilled operator and a relatively large machine limit the application of CDU. Handheld Doppler (HHD) is another popular technique for the identification of perforators; however, owing to its disability of visualization and high dependence on the vascular anatomy [11], it also remains controversial.

Recently, dynamic infrared thermography (DIRT) has attracted the attention of scientists because of its successful mapping of perforators thereby facilitating flap design optimization and assessing blood flow from perforators [12]. It appears to be an ideal alternative technique to identify perforators [13,14]. A previous study indicated that the integration of DIRT with another approach might enhance the accuracy of perforator localization [15]. Recently, infrared cameras have become small, affordable, and smartphone-compatible allowing their wider use for DIRT. For example, the FLIR ONE PRO, a smartphone-compatible infrared camera with a small bulk and low cost, has been used to detect perforators in the anterior abdominal wall [16]. Therefore, we investigated whether the combined application of HHD and the FLIR ONE PRO can improve the value of DIRT for mapping cutaneous perforators.

However, some challenges remain for perforator selection in flap surgery, such as flap thickness at the location, pedicle length, and intramuscular course. In ALTP flaps, the preoperative prediction of perforators based on the descending branch of the LCFA is another challenge because a careless design of the flap around the pedicle with anatomical variation can lead to difficult separation, a short pedicle, or other problems. Because specific rewarming patterns are caused by different blood flow intensities, the FLIR ONE PRO may be able to predict perforators according to different blood flow intensities. Thus, this study aimed to explore whether the combined application of HHD and FLIR ONE PRO imaging can improve the value of DIRT in mapping cutaneous perforators and whether the FLIR ONE PRO can predict perforators from the descending branch of the LCFA.

## 2 Methods

### 2.1 Patients

The patients in our study were recruited from the Department of Microsurgery and Hand Surgery in the First Affiliated Hospital of Sun Yat-sen University and the Fourth Department of Trauma and Hand Surgery in Guangzhou He Ping Orthopedic Hospital from March 15, 2021 to January 2, 2023. The inclusion criteria were as follows: 1) patients who agreed to participate in this study and 2) patients who underwent extremity microsurgical reconstruction using free perforator flaps. The exclusion criteria were as follows: 1) history of local thigh surgery; 2) history of vasculitis; 3) body mass index (BMI) > 30 or < 16 kg/m^2^; 4) febrile disease; 5) history of cardiovascular risk factors, including smoking, hypertension, diabetes mellitus, and dyslipidemia; 6) age >70 years; 7) history of cardiovascular and cerebrovascular diseases; and 8) history of excessive drinking. In this study, patients older than 45 years were grouped as elderly, and those younger than 45 years were grouped as young. The study protocol ([2021]386) was approved by the Institutional Review Board of the First Affiliated Hospital of Sun Yat-sen University, and written informed consent was obtained from all participants for the publication of their images. This study was conducted in accordance with the Ethics Code of the World Medical Association (Declaration of Helsinki).

### 2.2 Detection of perforators

**Fig. 1:**
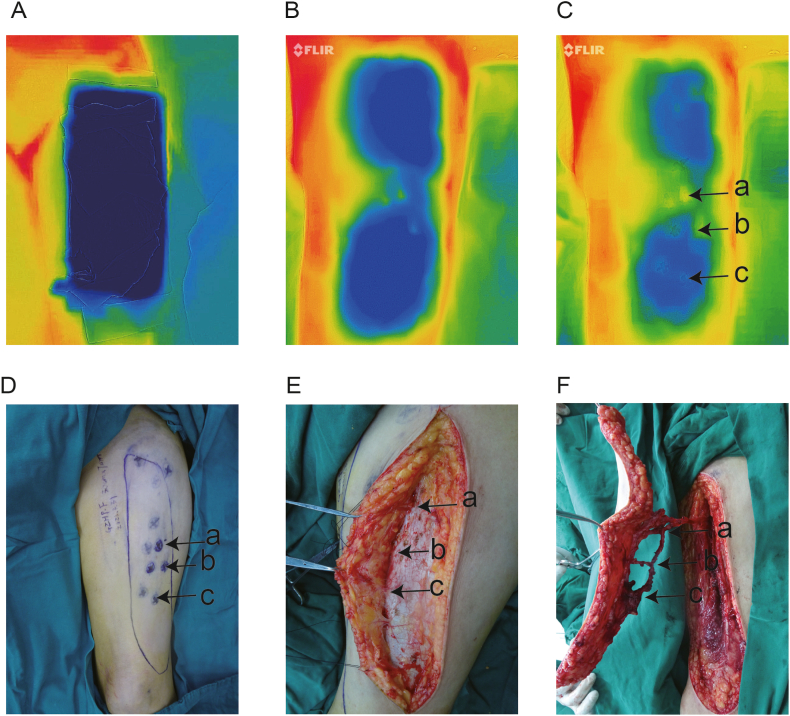
Locating the perforators of the ALTP flap in case 6. (A) Infrared image during the cold challenge of the ALTP flap. (B) The hotspots in an infrared image at 0 s. (C) The hotspots in an infrared image at the moment when all hotspots were vivid. (D) Preoperative mapping on the donor site. (E, F) Intraoperative identification of the ALTP flap perforators. The labels “a,” “b,” and “c” represent the first, second, and third perforator, respectively, from the descending branch of the lateral circumflex femoral artery (LCFA). “⊕” shows the hotspots marked by the FLIR ONE PRO camera, and “⊕·” shows the hotspots marked by FLIR ONE PRO + HHD. ALTP flap, anterolateral thigh perforator flap.

A 1-min cold challenge was performed using a reusable cold/hot gel pad (Lan Luo, China) with dimensions of 10 × 30 cm. The gel pad was pre-frozen at a temperature of −20 °C until the gel changed to a solid-liquid mixture. Patients were placed in a supine position in a room at 22 °C. The FLIR ONE PRO camera (resolution: 160 × 120 pixels and noise equivalent temperature difference of 150 Mk, 0.15 °C; T06KC100171, FLIR Systems, USA) was connected to an iPhone XR (Apple Inc., Cupertino, California) and mounted on a tripod approximately 50 cm from the region of interest. Next, the region of interest was exposed, and a cold challenge was conducted on the skin of the region of interest for 1 min, centered at the midpoint of each flap (Fig. 1A and B). Infrared thermography images were captured using the FLIR ONE PRO camera 40 s from the beginning of rewarming, the time when all hotspots were vivid (Fig. 1C). Specific spots on the skin that showed a faster pattern of rewarming compared with the surroundings were defined as hotspots [17]. During the recovery session, the hotspots imaged using the FLIR ONE PRO camera were included in the FLIR ONE PRO group. The existence of perforators under the hotspots was further confirmed by detecting blood flow sounds using HHD ultrasound (ES-101EX, Hadeco, Japan). Hotspots that were detected by both HHD ultrasonography and the FLIR ONE PRO camera were included in the FLIR ONE PRO + HHD group. The position, shape, and size of the flaps were designed based on the patients’ limb defects. Identified perforators in the subcutaneous layer were confirmed when the flaps were lifted during the operation (Fig. 1D and E). Dominant perforators were used to return retrogradely to the source artery to extend the vascular pedicle (Fig. 1F). A location was considered successful if the distance between the center of the hotspot and the subcutaneous dot with which the perforator was connected was <1 cm. Perforators with an external diameter <0.5 mm and those outside the planning region were excluded from subsequent statistical analyses. The procedure for perforator identification in our study remained the same as planned in all cases. Fig. 1 shows the entire procedure of perforator location in an ALTP. The method for investigating other regions was the same as that used for ALTP.

### 2.3 Differentiation of perforators based on the descending branch of the LCFA

The infrared images of the ALTP containing hotspots were further analyzed using FLIR Tools (FLIR Systems, Wilsonville, Oregon), a specific application for analyzing infrared images obtained using the FLIR ONE PRO. It has been reported that the first perforator (FP) is located at the midpoint of the A-P line (a straight line connecting the anterior superior iliac spine and the superolateral corner of the patella). Therefore, we recorded the central temperatures of the hotspots at a distance of <10 cm from the midpoint in each ALTP flap. The hotspots with the highest temperatures were regarded as the FPs of the descending branch of the LCFA. In addition, we identified FPs during the operation using the same method mentioned above.

### 2.4 Statistical analysis

**Fig. 2:**
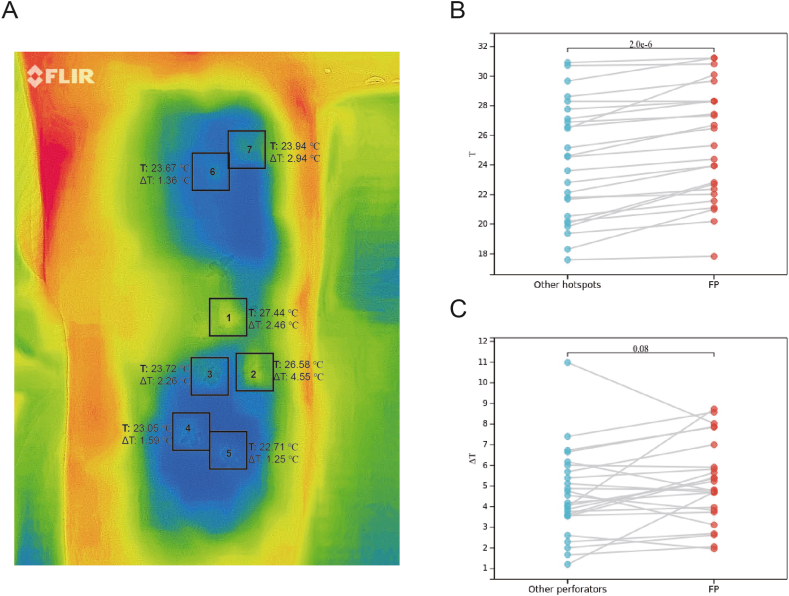
Analysis of rewarming patterns by FLIR Tools. (A) Analysis of temperature characteristics of the first perforator and second perforator of the descending branch of the LCFA in case 6. Boxes 1–7 show the hotspots around the midpoint of the A-P line. (B) Comparison between T of the first perforator (FP) and the highest T among other perforators. (C) Comparison of ΔT of FP and the highest ΔT among other perforators. T, temperature of a hotspot. ΔT, difference between the temperature of a hotspot and that of its surrounding. A-P line, a straight line connecting the anterior superior iliac spine and the superolateral corner of the patella.

Age, BMI, and temperature are shown as mean ± standard deviation. Patients aged ≤45 years were defined as the young group (n = 10), and those aged >45 years were defined as the elderly group (n = 12). The positive predictive values and sensitivities of the FLIR ONE PRO and FLIR ONE PRO + HHD groups were calculated. “T” represents the highest temperature of a hotspot, whereas “ΔT” represents the difference between the highest temperature of a hotspot and the lowest temperature of its surroundings, automatically recognized by FLIR Tools (Fig. 2A). The T and ΔT values of perforators from the descending branch of LCFA were analyzed by Wilcoxon matched-pairs signed rank test (Sangerbox 3.0). All other data were analyzed using GraphPad Prism 7 software. Statistical significance was set at p < 0.05.

## 3 Results

### 3.1 Clinical characteristics of enrolled participants

A total of 22 patients (7 women and 15 men) with extremity defects were enrolled in our study (Table 1). The mean age of these study participants was 44.6 ± 11.8 years (range 16–68 years), and their mean BMI was 22.0 ± 1.5 kg/m^2^. In these patients, 29 flaps, including 26 ALTP flaps, 1 peroneal artery perforator (PAP) flap, 1 superficial circumflex iliac artery perforator (SCIAP) flap, and 1 posterior interosseous artery perforator (PIAP) flap, were obtained. Among these, 14 and 12 ALTP flaps were harvested from the left and right thighs, respectively. Both PAP and PIAP flaps were from the left limbs, and the SCIAP flap was from the right lower abdomen.

**Table 1:** The characteristics of subjects and flaps.

No. of cases	Sex	Age (years)	BMI (kg/m^2^)	Flap	Donor sides	No. of flaps
1	M	16	20.02	ALTP	left, right	2
2	M	49	24.01	ALTP	left	1
3	M	35	22.41	ALTP	right	1
4	F	47	20.69	ALTP	right	1
5	M	40	23.23	ALTP	left	1
6	F	48	19.98	ALTP	left, right	2
7	M	32	25.10	ALTP	left, right	2
8	M	46	23.05	ALTP	right	1
9	M	27	22.65	ALTP	left	1
10	M	46	22.31	PAP	left	1
11	M	46	21.77	ALTP	left, right	2
12	M	40	23.53	ALTP	right	1
13	F	68	20.03	ALTP	right	1
14	M	34	21.63	ALTP	left	1
15	M	41	21.97	ALTP	left, right	2
16	M	60	23.31	ALTP	right	1
17	M	44	23.18	ALTP, SCIP, PIAP	left: ALTP, PIAP right: SCIP	3
18	M	44	21.48	ALTP	left	1
19	F	63	19.23	ALTP	left	1
20	F	49	21.88	ALTP	left	1
21	F	58	21.10	ALTP	right	1
22	F	48	21.19	ALTP	left	1

“M” and “F”: male and female; “ALTP”: anterolateral thigh perforator; “PAP”: peroneal artery perforator; “PIAP”: posterior interosseous artery perforator; “SCIP”: superficial circumflex iliac artery perforator; “BMI”: body mass index.

### 3.2 Evaluation of FLIR ONE PRO + HHD for the localization of perforators

The study results showed that 131 hotspots were visualized using FLIR ONE PRO, and 119 hotspots were further confirmed using HHD. Finally, 106 perforators were identified in the subcutaneous fascia and fat layers during surgery. The details of the perforators used for detection are presented in Table 2.

**Table 2:** Number of perforators identified by FLIR ONE PRO, HHD and during operation.

Case No.	No. in FLIR ONE PRO group	No. in FLIR ONE PRO + HHD group	No. identified intraoperatively
1	L 3; R 2	L 3; R 2	L 3; R 2
2	L 3	L 3	L 2
3	R 3	R 3	R 3
4	R 13	R 12	R 10
5	L 3	L 3	L 1
6	L 9; R 4	L 7; R 4	L 7; R 4
7	L 6; R 5	L 5; R 5	L 3; R 7
8	R 1	R 1	R 1
9	L 3	L 2	L 2
10	L 4	L 2	L 5
11	L 12; R1	L 11; R1	L 6; R1
12	R_PAP_ 2	R_PAP_ 2	R_PAP_ 2
13	R 10	R 9	R 6
14	L 5	L 5	L 6
15	L 11; R 6	L 9; R 6	L 7; R 6
16	R 3	R 3	R 3
17	L 3; L_PIA_ 2; R_SCIP_ 1	L 3; L_PIA_ 2; R_SCIP_ 1	L 2; L_PIA_ 2; R_SCIP_ 1
18	L 1	L 1	L 1
19	L 1	L 1	L 1
20	L 4	L 4	L 5
21	R 6	R 5	R 4
22	L 4	L 4	L 4

“L”or “R”: the number of perforators in the left ALTP flaps or the right ALTP flaps; “R_PAP_”: the number of peroneal artery perforators on the right side; “L_PIA_”: the number of posterior interosseous artery perforators on the left side; “R_SCIP_”: the number of superficial circumflex iliac artery perforators on the right; “HHD”: hand-held Doppler ultrasound; “No.“: number of perforators; ALTP: anterolateral thigh perforator.

To evaluate the use of FLIR ONE PRO + HHD in the localization of perforators in all study participants, we calculated the sensitivity and positive predictive value (Table 3). The results showed that in all study participants, the sensitivity of FLIR ONE PRO + HHD (95.28%) was lower than that of the FLIR ONE PRO (97.17%), whereas the positive predictive value of FLIR ONE PRO + HHD (84.87%) was higher than that of the FLIR ONE PRO (78.63%). Next, we considered the differences in blood perfusion in the young and the elderly [18] and evaluated the value of FLIR ONE PRO + HHD in 10 young patients (age ≤45 years) and 12 elderly patients (age >45 years; Table 3). According to the results, FLIR ONE PRO + HHD showed a sensitivity of 97.87% and a positive predictive value of 88.46% in the young group and a sensitivity of 93.22% and a positive predictive value of 82.09% in the elderly group. These data showed that the combined application of FLIR ONE PRO and HHD increased the positive predictive value in both young and elderly groups; however, in the elderly group, the sensitivity was decreased.

**Table 3:** The effect of FLIR ONE PRO + HHD on the perforator prediction.

			Intra-operative Findings	
Methods	Positive/Negative	Age (years)	Positive	Negative
FLIR ONE PRO	Positive	<45	46	10
		>45	57	18
		total	103	28
	Negative	<45	1	–
		>45	2	–
		total	3	–
FLIR ONE PRO + HHD	Positive	<45	46	6
		>45	55	12
		total	101	18
	Negative	<45	4	–
		>45	1	–
		total	5	–

FLIR ONE PRO: control group; FLIR ONE PRO + HHD: FLIR ONE PRO + HHD group; HHD: hand-held Doppler ultrasound.

### 3.3 Rapid prediction of perforators derived from the descending branch of the LCFA

Next, we explored whether FLIR Tools can rapidly predict the FP based on the descending branch of the LCFA among the 116 hotspots at a distance <10 cm from the midpoint of the A-P line in the 26 ALTP flaps (Fig. 2). We used FLIR Tools to determine the hotspot region with the highest center temperature, namely FP, from the descending branch of the LCFA. The results showed that the sensitivity, specificity, positive predictive value, and negative predictive value were 96.15%, 98.9%, 96.15%, and 98.9%, respectively (Table 4). Next, we selected the FP based on the descending branch of the LCFA in each ALTP flap and the hotspot with the highest T among the remaining hotspots for subsequent analyses. The results showed that the T of FPs was 25.28 ± 3.8 °C at 40 s after rewarming beginning, which was higher than that of the hotspots with the highest T (24.21 ± 3.98 °C, p < 0.05; Fig. 2B). The identification of FPs was completed in less than 5 min. In addition, a higher ΔT was beneficial for differentiating hotspots using the FLIR ONE PRO, and we analyzed the ΔT of FPs and hotspots with the highest T in each flap. According to FLIR Tools (Fig. 2C), the ΔT of FPs at 40 s after rewarming beginning was 5.10 ± 1.97 °C, which was higher than the mean ΔT of the hotspots with the highest T (4.59 ± 2.09 °C), but there was no significant difference (P > 0.05). Interestingly, our results indicated that the use of the FLIR ONE PRO helps to rapidly differentiate FPs of the descending branch of the LCFA from others in the ALTP flap based on the center temperature of the hotspots.

**Table 4:** The effect of FLIR ONE in location of FP of the descending branch of LCFA.

		Intra-operative finding	
		Positive	Negative
**FLIR ONE PRO**	Positive	25	1
	Negative	1	90

## 4 Discussion

Perforator flaps are the most commonly used technique in traumatology and microsurgery. Successful preoperative localization of the perforators reduces the time and difficulty of perforator flap surgery. However, the anatomy of the perforators is diverse among patients. Therefore, scientists have been conducting studies to provide a low-cost, noninvasive, and rapid method that can locate perforators preoperatively [19].

Recently, DIRT has appeared to be an ideal alternative for other predictive technologies in perforator localization owing to its potential for visualizing perforators noninvasively and at low costs [20]. Researchers have used this technique to preoperatively detect the human deep inferior epigastric artery [21]. Because the ALTP flap is the most frequently used method for external defects [22], Xiao et al. explored the effects of DIRT on the preoperative location of perforators in ALTP flaps and found that the accuracy of a high-resolution infrared camera was close to that of CDU [23]. Color-coded duplex sonography has been proven to be useful for identifying microvessels because of its 100% sensitivity, positive predictive value, and accuracy [24]. Compared with color-coded duplex sonography, the FLIR ONE PRO is a new infrared camera that is more convenient, affordable, and portable. It achieves perforator localization based on thermal imaging.

Previously, it was suggested that the FLIR ONE PRO is a useful tool for the detection of perforators with high sensitivity and specificity [13,25]. Nevertheless, another study indicated that it should be used as an adjunct tool, along with established imaging techniques [17]. Although HHD ultrasound cannot visualize the vasculature, it remains popular and is widely used to locate perforators nowadays [26]. Furthermore, the positive predictive values of HHD differed significantly among studies [[27], [28], [29]], which might be the result of the diversity of the vascular anatomy. However, our results showed that the combined application of the HHD could optimize the perforator localization using the FLIR ONE PRO because the FLIR ONE PRO + HHD group showed an increased positive predictive value (84.87%), although the sensitivity was slightly decreased (95.28%), possibly because of the increased marginal benefit. However, an increase in the positive predictive value is important for a surgeon. Therefore, compared with the use of the FLIR ONE PRO alone, the combined use of HHD and FLIR ONE PRO seems to be more valuable in preoperative perforator localization.

With increasing age, both thermoregulation ability and surface temperature of the skin decline [18,30]; therefore, the mapping of perforators by the FLIR ONE PRO may be more difficult in the elderly. According to our study, the FLIR ONE PRO + HHD approach showed a higher sensitivity and positive predictive value in the young group than in the old group. Therefore, the age of the participants may be a crucial factor that influences the FLIR ONE PRO + HHD efficacy. Based on our study, we suggest the combined application of FLIR ONE PRO and HHD in both young and older patients because it can increase the positive predictive value, which is an important index for surgeons to avoid incorrect tissue separation.

The dominant perforator is selected to supply the flap as it provides the best chance for flap survival [31]. Perforators derived from the descending branch of the LCFA are frequently considered the dominant option for supplying an ALTP flap; however, they may have several anatomic variations in terms of number, size, and course [4]. Some studies have counted 1.66 ± 0.70 perforators from the LCFA per extremity, with an average of 5.38 cm between adjacent perforators [32]. However, it is difficult to quickly and accurately distinguish FPs from other perforators based on the descending branch of other perforators. In our study, we used the FLIR ONE PRO to accurately predict the FP of the descending branch of the LCFA within 5 min based on the highest temperature of the hotspot center. Thus, it was possible for the FLIR ONE PRO to rapidly locate the perforators of the descending branch of the LCFA because these perforators have a better blood supply. However, according to the manufacturer, the FLIR ONE Pro has an accuracy of ±3 °C or ±5%, which suggests that the error between the actual temperature and the measured temperature of the hotspot is ±3 °C or ±5%. To ensure that T and ΔT in our results are close to the true values, we should maintain consistency in the measuring angles, ambient temperature, air quality, and, especially, the distance from the measured regions.

This study has some limitations. First, the intraoperative validation of the perforators was performed by two different teams of surgeons, although the preoperative examination was conducted by the same person. Second, the specificity could not be calculated because of the absence of true negative outcomes. Third, capillary-type perforators were excluded from our study because of the difficulty in their visualization using the FLIR ONE PRO [33]. Fourth, we didn't enroll young females affected by serious soft tissue defects in our study, because of the low incidence of serious trauma in them. Fifth, the use of a cold/hot gel pad can lead to skin artifacts and irregular cooling patterns. Although most researchers have used a cold/hot gel pad to cool flaps and then image hotspots via an infrared camera, this might lead to artifacts on the skin and irregular cooling patterns. In future studies, we should perform precooling via airflow followed by alcoholic disinfection, which provides the clearest thermograms and fastest results [34]. Finally, the sample size of our study was small, and the small sample size might influence the results in the young and elderly groups. Thus, a larger sample size is required to further verify our findings.

In this study, we investigated the value of the FLIR ONE PRO in predicting the location of perforators. The results showed that the combination of FLIR ONE PRO and HHD can increase the positive predictive value of preoperative perforator localization but may slightly decrease sensitivity. The combined use of the FLIR ONE PRO and HHD at the perforator location was more effective in young than in elderly patients. Additionally, we found that the FLIR ONE PRO can rapidly differentiate the perforators of the descending branch of the LCFA from other perforators.

## Author contribution statement

Weiwen Zhu, Yi Yang, Jiyong Jiang, Ping Li, Ming Fu: Conceived and designed the experiments; performed the experiments; analyzed and interpreted the data; contributed reagents, materials, analysis tools, or data; and wrote the manuscript.

Qingtang Zhu, Jian Qi: Performed the experiments; analyzed and interpreted the data.

Bengang Qin, Jingyuan Fan: Conceived and designed the experiments; analyzed and interpreted the data; contributed reagents, materials, analysis tools, or data.

## Ethics statement

The study protocol ([2021]386) was approved by the Institutional Review Board of the First Affiliated Hospital of Sun Yat-sen University, and written informed consent was obtained from all participants. This study was conducted in accordance with the Ethics Code of the World Medical Association (Declaration of Helsinki).

## Data availability statement

Data will be made available on request.

## Additional information

No additional information is available for this paper.

## Declaration of competing interest

The authors declare that they have no known competing financial interests or personal relationships that could have appeared to influence the work reported in this paper.
